# The Isolation of DNA by Polycharged Magnetic Particles: An Analysis of the Interaction by Zeta Potential and Particle Size

**DOI:** 10.3390/ijms17040550

**Published:** 2016-04-20

**Authors:** Yazan Haddad, Kledi Xhaxhiu, Pavel Kopel, David Hynek, Ondrej Zitka, Vojtech Adam

**Affiliations:** 1 Department of Chemistry and Biochemistry, Faculty of Agronomy, Mendel University in Brno, Zemedelska 1, CZ-613 00 Brno, Czech Republic; yazanhaddad@hotmail.com (Y.H.); paulko@centrum.cz (P.K.); d.hynek@email.cz (D.H.); zitkao@seznam.cz (O.Z.); 2 Central European Institute of Technology, Brno University of Technology, Technicka 3058/10, CZ-616 00 Brno, Czech Republic; 3 Department of Chemistry, Faculty of Natural Sciences, University of Tirana, Blv. Zog I, No. 2/1, 1001 Tirana, Albania; kledi.xhaxhiu@unitir.edu.al

**Keywords:** magnetic, nanoparticles, DNA isolation, zeta potential, particle size

## Abstract

Magnetic isolation of biological targets is in major demand in the biotechnology industry today. This study considers the interaction of four surface-modified magnetic micro- and nanoparticles with selected DNA fragments. Different surface modifications of nanomaghemite precursors were investigated: MAN37 (silica-coated), MAN127 (polyvinylpyrrolidone-coated), MAN158 (phosphate-coated), and MAN164 (tripolyphosphate-coated). All particles were positive polycharged agglomerated monodispersed systems. Mean particle sizes were 0.48, 2.97, 2.93, and 3.67 μm for MAN37, MAN127, MAN164, and MAN158, respectively. DNA fragments exhibited negative zeta potential of −0.22 mV under binding conditions (high ionic strength, low pH, and dehydration). A decrease in zeta potential of particles upon exposure to DNA was observed with exception of MAN158 particles. The measured particle size of MAN164 particles increased by nearly twofold upon exposure to DNA. Quantitative PCR isolation of DNA with a high retrieval rate was observed by magnetic particles MAN127 and MAN164. Interaction between polycharged magnetic particles and DNA is mediated by various binding mechanisms such as hydrophobic and electrostatic interactions. Future development of DNA isolation technology requires an understanding of the physical and biochemical conditions of this process.

## 1. Introduction

Bionanotechnology is a new interdisciplinary area that integrates biotechnology and nanotechnology; two of the 21st century’s most promising technologies. This integration provides an unprecedented opportunity to develop new tools for medical, agricultural, and environmental applications. Understanding the mechanisms of binding of nucleic acids on nanoparticles is important for various applications such as molecular detection and gene delivery [1,2]. Research on nanoparticle interaction with DNA has started to explore the applications of carbon nanoparticles [3], silica nanoparticles [4], gold nanoparticles [5], silver nanoparticles [6], and iron/magnetic nanoparticles [7]. Carbon nanoparticles were previously shown to bind Lambda phage DNA [3]. Carbon nanomaterials have been reported to inhibit DNA associated enzymes, e.g., blocking the polymerase chain reaction [1]. However, with proper surface modifications, it is possible to produce suitable carbon nanomaterials for medical applications [8]. Silica-based nanoparticles can be used either directly or with modified surfaces (e.g., amino-coated or specific oligonucleotide-coated) to electrostatically bind, condense, and protect DNA from cleavage [9]. Kneuer *et al.* investigated binding of plasmid DNA on silica nanoparticles coated with positively charged functional groups [4]. Furthermore, single stranded DNA was found to form a stronger binding with silica when compared to double-stranded DNA [10]. Gold nanoparticles were also shown to bind single stranded DNA with higher affinity than double-stranded DNA [5], which qualifies gold nanoparticles for use with oligonucleotide probes to specifically isolate DNA sequences [11]. Unmodified silver nanoparticles were shown to bind single nucleobases. This interaction was characterized with a change in nanoparticle color, correlating with aggregation and the type of nitrogen base [6]. Silver nanoparticles modified with oligonucleotides are ultrasensitive for detection of DNA [12]. Magnetic nanoparticles are more convenient for isolation and immobilization of biological materials due to the versatile choices of coating through chemical modification and addition of functional groups. The modification of magnetic nanoparticles with enzymes, proteins, and DNA is currently used in medical immunoassays and now suggested for magnetically controlled target delivery of anticancer drugs [13].

Magnetic isolation is one of the most effective applications of magnetic particles. Suitably modified nanoparticles efficiently separate ions, pollutants, cells, DNA, proteins/antibodies, and various bioactive substances. Several ready-to-use magnetic particles are commercially available such as: magnetic Sepharose^®^, Dynabeads^®^, and Chemicell^®^. Magnetic micro- and nanoparticles are cheap, recyclable, versatile, fast, and compatible with heterogeneous biological solutions [14]. Magnetic isolation is the application of using magnetic beads or particles that are biologically or chemically modified to interact with a specific target. After mixing the magnetic particles and the solution containing the target, targets bound to particles are separated; a permanent magnet is used to align atomic dipoles of the particles to induce a magnetic field in the solution [15]. Pershina *et al.* provide a comprehensive review of the literature regarding magnetic nanoparticle binding of DNA [7]. A great variety of magnetic particles have been developed with adjustable physicochemical properties in bio-relevant media (e.g., size, magnetic moment, surface charge, morphology, shell thickness), taking into account protection, stabilization, and functionalization of magnetic nanoparticles by designed coating [16]. The spherical shape and reduction in particle size result in decreased magnetic properties, while modifying agents (organic or inorganic) can be used to form a protective surface layer (either electrostatic or covalent) with a thickness in the range of 1–5 nm for small modifying molecules and up to more than 100 nm for large polymers [17]. The composition and coating of nanoparticle surface, and binding conditions are among the major highlights of research in this field. Proper coating of magnetic particles is important to prevent aggregation of particles in multi-core structures [18]. Monodispersed single-core magnetic particles usually have more uniform properties, while multi-core magnetic particles exhibit stronger magnetic properties.

In this work, four modifications of nanomaghemite particles were investigated: MAN37 (silica-coated), MAN127 (polyvinylpyrrolidone-coated), MAN158 (phosphate-coated), and MAN164 (tripolyphosphate-coated). DNA isolation with magnetic particles was evaluated by quantitative PCR. The use of zeta potential and particle size provided valuable insight to DNA-particle interaction and to quantitative PCR evaluation.

## 2. Results

**Figure 1:**
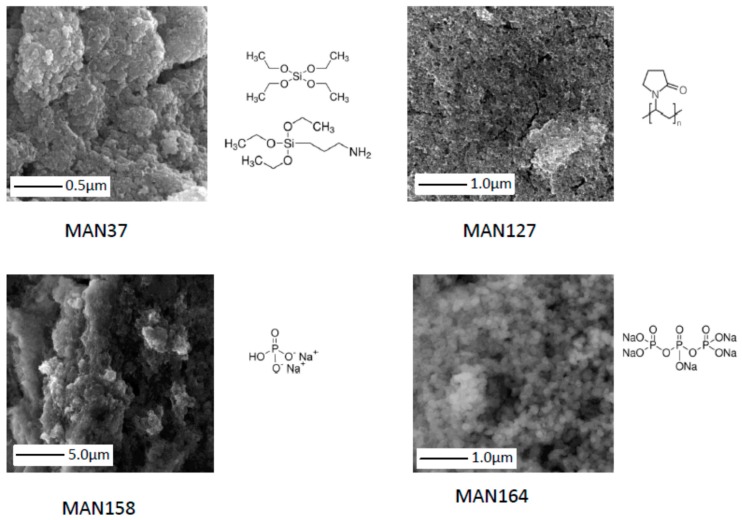
Scanning electron microscopy (SEM) of magnetic particles showing the external structure of the particles caused by chemicals used in modification of maghemite core. MAN37 (silica-coated), MAN127 (polyvinylpyrrolidone-coated), MAN158 (phosphate-coated), and MAN164 (tripolyphosphate-coated).

Magnetic micro- and nanoparticles were prepared by reduction of iron(III) nitrate by sodium borohydride in ammonia solution. Several coating modifications were applied to attain charged surfaces able to bind DNA fragments. Analysis by scanning electron microscopy (SEM) showed that magnetic particles prepared in this work were formed by agglomeration of spherical nanoparticles into multi-core irregular structures (Figure 1). However, further analysis by zeta potential and particle size measurement (as shown below) showed monodispersed and polycharged magnetic particles. The information obtained from both methods confirms the existence of a dynamic equilibrium between particles in such a system that determines the critical size and the respective charge of each agglomerate. It is assumed that for each type of surface modification, the process of agglomeration (particle formation) resulted in different size, shape, and charge according to chemical nature of modifying agent.

For testing of DNA isolation efficiency by magnetic particles, a binding solution of high salt content, acidic pH, and dehydration properties was used to precipitate a total of ~10^11^ copies of purified 700 base paired double-stranded DNA on magnetic particles. DNA/binding solution/particles were mixed and immobilized on magnet. After washing with ethanol, residual DNA was rehydrated and eluted with a pH-8 Tris-HCl buffer and then amplified using a quantitative PCR. PCR was performed in replica and achieved 0.999–1.000 *R*^2^ value for standard regression. The best amplification was observed in DNA eluted from MAN127 and MAN164 magnetic particles (Table 1). According to quantitative PCR, MAN127 eluted 4.17 × 10^9^ copies/μL, while MAN164 eluted 1.91 × 10^9^ copies/μL. These numbers were 100s times higher than the number of copies of DNA eluted from MAN37 and 1000s times higher than of number of copies of DNA eluted from MAN158.

All the magnetic particles displayed positive zeta potential in the range of 1.78–5.55 mV (Table 1), which is compatible with zeta potential of DNA in the binding solution (approx. −0.22 mV). The low positive zeta potential of particles accompanied with a comparative wide standard deviation indicates a polycharged system of agglomerated particles. The highest zeta potential was observed by silica-coated MAN37 and PVP-coated MAN127. The exposure to DNA lowered the measured zeta potential near to zero. In the case of polyphosphate-coated MAN164, the zeta potential reversed from +1.78 to −1.25 mV. In quantity, zeta potential of MAN37 decreased by 5.5 mV upon exposure to DNA, whereas zeta potential of MAN127 and MAN164 decreased by nearly 3 mV. Zeta potential results indicate that DNA-particle interaction occurred in MAN37, MAN127, and MAN164. This was confirmed by quantitative PCR for MAN127 and MAN164, but not for MAN37.

Data from electron microscopy (Figure 1) and zeta potential measurements (Table 1) indicate that the magnetic particles were non-uniform clustered (agglomerated) polycharged particles; however, the mathematical model used for estimation of particle size shows that they are classified as monodisperse systems. Particle size measurements showed significant increase when MAN164 particles were exposed to DNA. Particle size increased from 2.9 to 4.8 µm, indicating physical clustering of particles in presence of DNA. No observable change in particle size was observed for MAN37, MAN127, or MAN158 particles.

**Figure 2:**
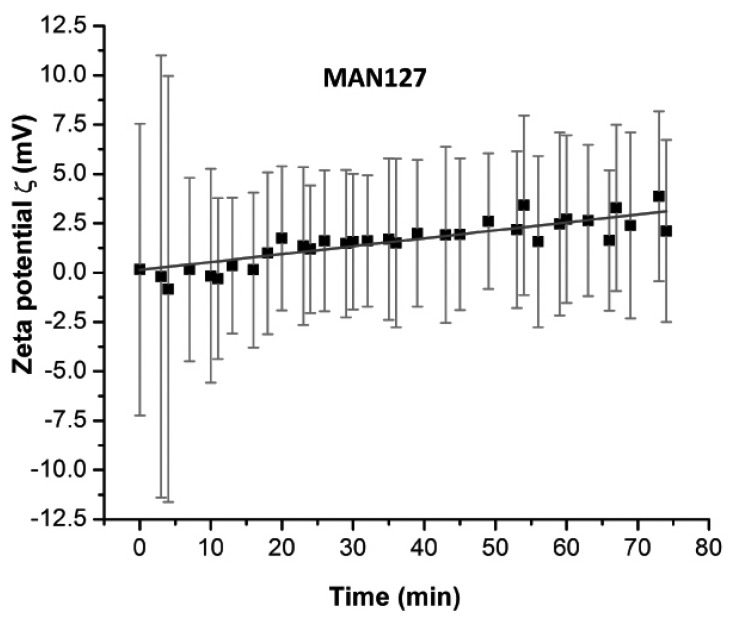
Zeta potential variation over time of MAN127 in presence of DNA.

To test the binding reaction in time, zeta potential readings were taken. Within the first five minutes, the standard deviation of zeta potential exceeded 20 mV before stabilizing to a range below 10 mV, as shown in Figure 2.

## 3. Discussion

The study of interactions between nanoparticles and DNA offers rich new information on the mechanisms that control and alter DNA molecular structure and highlights new insights for molecular detection and gene delivery [1,2]. Previous literature regarding magnetic nanoparticle binding to DNA has been comprehensively reviewed [7], and a variety of magnetic particles has been developed with adjustable physicochemical properties [16]. Modified magnetic micro- and nanoparticles are cheap, recyclable, versatile, fast, and compatible with heterogeneous biological solutions. Several ready-to-use magnetic particles are now commercially available [14]. In this study, four modified magnetic particles were synthesized and screened for their potential to bind and elute DNA. Silica, PVP, phosphate, and polyphosphate were used to coat magnetic particles to give their surface ionic charge.

MAN37 are coated by TEOS and 3-aminopropyl triethoxysilane. Silica-based coating has been investigated in the past due to their decreased toxicity, their chemical stability, and their charge that contributes to high electric coulomb repulsion of magnetic nanoparticles [19]. This method, also known as the Stöber process, directly coats magnetic particles with amorphous silica because of the strong affinity of iron towards silica without the need for a linker to promote the deposition of silica [20]. The silanol groups of silica surface are often covered with one or more layers of hydrogen bound water molecules that can be disrupted in the presence of high ionic strength salts (*i.e.*, chaotropic ions). Chaotropic ions cover the particle surface with ionic charges and hydrophobic functional groups. The mechanism of DNA interaction with silica involves short-range phosphate-silanol interactions and juxtaposed hydrophobic functional groups. These bonds supported by the electrical double layer are strong enough to overcome the repulsion between negative charges of DNA and silica. In other words, both DNA and silica carry functional groups that are charged or hydrophobic; so binding is only a matter of alignment of complementing functional groups [10,21].

MAN127 were coated by modification with polyvinylpyrrolidone (PVP). PVP is known for its long standing safety of use as a low viscosity coating material in biomedical and pharmaceutical applications [22,23]. PVP has been previously applied as a separation matrix for DNA due to its sieving abilities [24]. A study on the use of PVP as a surface coating during the process of PCR showed that, if PVP interacts with DNA, such an interaction does not inhibit PCR [25], supporting the hypothesis that, even though hydrophobic interaction is involved, it might not affect the hydrogen bonding or hybridization of nitrogen bases.

MAN158 were chemically modified with sodium phosphate, whereas MAN164 were modified with sodium tripolyphosphate where low soluble calcium tripolyphosphate is produced. On the molecular level, previous studies suggest that phosphonate and phosphate ions form bidentate interactions with particle surface; with either one or two oxygen atoms of the phosphate group acting as clamps on iron oxide exterior [19].

High ionic strength and low pH conditions enhance thenegative charge of DNA and saturate the surface of magnetic particles with counter charge and counter hydrophobic areas. DNA-particle interaction was confirmed by the decrease in zeta potential from positive to negative or near zero for MAN37, MAN127, and MAN164 (Table 1). All considered magnetic microparticles revealed positive zeta-potentials, while the DNA fragments in solution were slightly negatively charged, causing electrical interaction between them. Here, the attachment of DNA fragments to all of the magnetic particles lowers their zeta potential, but at the same time ensures particle protection due to the particle surface coverage. Zeta potential characterization of all samples showed polycharged particles, with silica-coated and PVP-coated particles revealing the highest initial zeta potentials. Due to the interaction with DNA, the zeta potential decreased to near zero for MAN37 and MAN127 particles; meanwhile, in the case of MAN164, the zeta potential decreased to even lower than zero. It is important to address colloid stability with further studies. In cases of near zero, zeta potential of particles the monodisperse systems will be unstable. We believe this issue should be addressed in the development process of nanoparticles. Previously, Bagwe *et al.* showed that modifying silica nanoparticles particles with functional groups should be in optimized ratios between inert (e.g., methyl phosphonate) and active (e.g., amino) groups to obtain highly negative zeta potential and a well dispersed system [26]. The same concept applies to positively charged nanoparticles. In general, the ratio between inert/hydrophobic functional groups and charged functional groups is a major determinant to colloidal stability of particles in solution.

When DNA isolation efficiency was tested using quantitative PCR, salt was washed with ethanol after binding because it can inhibit molecular analysis, *i.e.*, polymerase enzymes. DNA was then eluted by using fully hydrated low ionic strength solution and high pH. Only MAN127 and MAN164 eluted high detectable amounts of DNA (Table 1). When the dilution factor was taken into consideration, binding percentage from the initial DNA amount (10^11^ copies) was 0.6%, 0.1%, and 76.5% for MAN37, MAN158, and MAN164, respectively. MAN127 binding percentage was 166.9%. These numbers reflect the variation in PCR process due to chemical enhancers and/or inhibitors [27]. Quantitative PCR confirmed the binding of DNA results from zeta potential experiment (Table 1) in the cases of MAN127 and MAN164, but not in the case of MAN37.Based on zeta potential data, MAN37 binds DNA in a very weak fashion that was easily washed by ethanol or that MAN37 might release chemicals that inhibit polymerase activity in PCR. On the other hand, traces of MAN127 particles and perhaps also MAN164 possibly worked as catalysts for the polymerase reaction. Previous literature reported enhancement of PCR in the presence of pyrrolidones at various concentrations; particularly, 2-pyrrolidone showed high potency, while polyvinylpyrrolidone was reported to enhance PCR by chelating polyphenolic inhibitors in reaction [28,29]. However, it is unclear whether polyphosphates might play role in enhancement or catalysis of polymerase chain reaction. The possibility of the release of chemicals that can inhibit PCR from the magnetic particles was not ignored. Several washing steps of particles on magnets were performed to avoid this as much as possible before the experiment and to ensure that only magnet-binding particles were retained. Mechanisms for inhibition of polymerase have been previously reported and several inhibitors have been identified. The polymerase enzyme can be degraded, denatured, or inhibited by blocking the active site. A high amount of divalent ions (Ca^2+^ and Mg^2+^) are more inhibitory than monovalent ions (K^+^ and Na^+^) [21,27].

The particle size measurements shown in Table 1 exhibited a considerable increase in MAN164 particles upon DNA exposure, leading to the conclusion of a significant binding efficiency of DNA fragments, which led to further particle agglomeration. As DNA accumulated on the particle surface, it is possible that particles started to cluster in pairs resulting in the double size increase.

Since MAN37 particle agglomerates are smaller in size compared to the other particles considered agglomerates, they exhibit a higher surface area as a consequence. Therefore, certain amounts of DNA fragments attached to MAN37 particles decrease their zeta potential more than in the case of bigger particles. Due to this fact, the decrease of zeta-potential for MAN37 particles was more significant than for all the others (Table 1). Anyway, since MAN37 particles surface can accommodate much fewer DNA fragments than other particles, their increase in size was not significant when compared to the other particles. Since MAN127 and MAN164 had similar sizes but different zeta potentials and behaved differently (Table 1), it seems that the particle size alone does not play the greatest role in the DNA fragment binding. A crucial factor that influences the zeta potential and the binding in this process is the particle surface modification, but also the respective double layer. Despite their initial size, the particle behavior was strongly influenced by their surface modification and consequently by the electrical double layer. MAN37 showed the smallest sizes and almost no size difference in contact with DNA. On the other hand, MAN158 was distinguished for their initial high size compared to MAN127 and MAN158; nevertheless, they did not reveal significant particle size variations in contact with DNA. Only MAN164 particles displayed a significant change in size in contact with DNA (Table 1), suggesting a mechanism of a further aggregation of particles in the presence of DNA, which led to bigger agglomerates. The high standard deviation in particle size indicates that at least two particle structures could bind in a stable fashion in solution (Table 1).

The structure and composition of nanoparticles, the pH at which DNA and nanoparticles interacted, and the functionalization of the nanoparticle surface are among the major highlights of the research in this field [18]. Several of our tests indicated that the particles were polycharged in a manner that the charge configuration of particles in solution depended on the electrical double layer around them and changes upon the DNA fragment exposure. One example is the multiple measurements of zeta potential of particles with and without DNA. Upon the DNA binding, the particles were stabilized and turned from polycharged to monocharged. Our analysis showed that DNA interaction with magnetic particles was fast and stabilizing within five minutes at room temperature (Figure 2).

Zeta potential can vary dramatically with changing conditions in ionic strength, pH and viscosity (or dehydration). It is important to emphasize that the zeta potential value detected for DNA (−0.22 mV) was explicit for binding conditions reported in this work and the small size of used DNA product (~700 bp). Furthermore, since zeta potential of DNA threads was detected in the presence of low DNA quantities (<1 ng), this advocates that, due to the precipitation process in dehydration conditions, DNA began to exhibit colloidal properties (detectable by zeta potential) that can contribute to the isolation process. Ideally, isolated genomic DNA exhibiting sizes of greater than 50 Mbp will show a higher zeta potential. In order to increase the negative zeta potential of DNA in routine laboratory settings, precipitation can be enhanced by increasing the concentration of DNA or by adding carrier DNA of different species.

**Figure 3:**
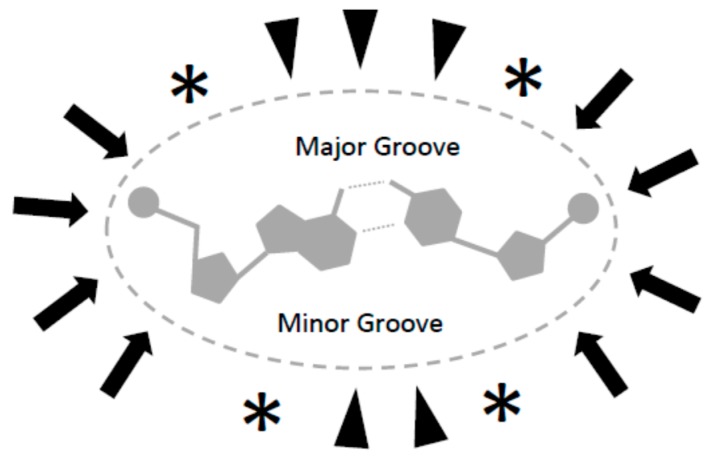
Cross section of a DNA molecule showing possible binding interactions with magnetic particles: electrostatic/ionic (**arrows**), hydrophobic (**stars**), and hydrogen donor/acceptors (**arrowheads**).

In dehydration conditions (e.g., in this experiment), the configuration change of DNA from B-form to shorter non-physiological precipitating A-form might affect the availability of binding contacts in the major and minor grooves of DNA [30]. Alternatively, zigzag Z-form DNA resulting from alternating purine-pyrimidine sequence suggests that DNA sequence itself might play a role in binding to magnetic particles. It is possible that several mechanisms play a role in the enhancement of particle-DNA binding in aqueous solution; such as hydrophobic interaction, coordinated hydrogen/dipole bonds interactions, and electrostatic/ionic interactions (Figure 3), in addition to physical mechanisms resulting from rough and irregular surfaces of particles, nucleic acid precipitation, aggregation, and physical torsion of DNA strands between multiple particles.

To understand the mechanisms of interaction between magnetic particles and DNA, more attention should be dedicated to the environmental conditions of this process (e.g., solvent, ionic strength, pH, and temperature) and to the structure of the modifying agents used for coating.

## 4. Experimental Section

### 4.1. Synthesis and Modifications of Magnetic Micro- and Nanoparticles

Nanomaghemite is prepared from iron nitrate and sodium borohydride in ammonia solution. 7.48 g of Fe(NO_3_)_3_·9H_2_O (Sigma-Aldrich, Saint Louis, MO, USA) was mixed in 400 mL of MilliQ water on a magnetic rotor. Separately, 1 g of NaBH_4_ was dissolved with 50 mL 3.5% NH_3_ solution for 10 min and then added to the iron nitrate solution. The resulting dark solution was boiled for 2 h and then left to mix overnight on a magnetic stirrer. Nanomaghemites were rinsed several times with MilliQ water and used for modifications.

MAN37: Maghemite was modified in a solution of isopropanol by tetraethyl orthosilicate (TEOS, Sigma-Aldrich, Saint Louis, MO, USA) and later by (3-aminopropyl)triethoxysilane (Sigma-Aldrich, Saint Louis, MO, USA) via a slightly modified method [31]. The product was rinsed several times on a magnet with diluted ethanol and then MilliQ water.

MAN127: Maghemite was mixed with a polyvinylpyrrolidone (PVP, 10k, 0.2 g, Sigma-Aldrich) solution and stirred overnight. The product was separated by magnet and rinsed several times with MilliQ water.

MAN158: Maghemite was stirred with a Na_2_HPO_4_·2H_2_O (1.2 M, 2 mL, Sigma-Aldrich) and Ca(NO_3_)_2_·4H_2_O (1 M, 4 mL, DuchefaFarma B.V., Haarlem, The Netherlands) solution at a 1:4 ratio for 2 h. Then, 1 M NaOH was added and stirred overnight. The product was separated by magnet and rinsed several times with MilliQ water.

MAN164: The surface of the prepared maghemite was modified by an addition of sodium tripolyphosphate pentabasic (Na_5_P_3_O_10_, Sigma-Aldrich, Saint Louis, MO, USA), followed by calcium nitrate (1 M, 4 mL, DuchefaFarma B.V., Haarlem, The Netherlands). Product was separated by magnet and rinsed several times with MilliQ water.

### 4.2. Scanning Electron Microscopy

Magnetic particles were visualized by scanning electron microscopy (SEM). Images of their external structure were taken using a MIRA II LMU (Tescan, Brno, Czech Republic) instrument equipped with an In-Beam SE detector. Abeam current of approximately 1.0 nA was used with accelerating 15,000 volts.

### 4.3. DNA Isolation Efficiency Test of Magnetic Particles Using Quantitative PCR

Large amounts of DNA were prepared using PCR to amplify a 700 bp product from the Ebola glycoprotein GP gene (EBOV subtype Zaire, strain Mayinga 1976). The following primers were used for amplification of Ebola DNA from the pCMV3-ZaireEBOV-U23187-GP-FLAG vector (Sino Biological Inc., Beijing, China): Forward: GACCCCCAAAAGCAGAGAAC. Reverse: ACGCCTGTAACTCCAATACCTG.

After the purification of DNA fragments using MinElute kit (Qiagen, Hilden, Germany), the concentration of DNA was first estimated using a nanodrop-based method on Infinite 200 PRO NanoQuant instrument (Tecan, Männedorf, Switzerland). Amounts of 100 ng/µL are equivalent to >10^11^ copies of DNA. The dilution of DNA to working solution of ~10^10^ copies/µL was amply found for all experiments. The same initial solution (10^10^ copies of DNA in Tris-HCl 10 mM pH = 8) was used for binding and as a standard in quantitative PCR. Binding mixture was prepared by adding 10 μL of DNA (~10^10^ copies/μL) to 10 μL of sodium acetate-HCl (0.75 M, pH = 6), and 60 μL of 85% ethanol to 8 μL of magnetic particles. After well mixing by pipetting or vortexing, the samples were set on magnet for 3 min. The solution was discarded, and magnetic particles were then washed with 100 μL of 85% ethanol while on magnet. DNA was eluted by changing the pH. Approximately 40 μL of Tris-HCl (10 mM; pH = 8) were used to elute the DNA after mixing and the tubes were then put on magnet for 3 min. Real-time PCR was used to quantitate the DNA using standards of 10^7^, 10^8^, 10^9^, and 10^10^ copies/µL. Standards were prepared by serial dilution of purified PCR product that was estimated using the nanodrop-based method. For quantitation of DNA, 20-µL PCR reaction mix was prepared using SYBR Green Quantitative RT-PCR Kit (Sigma-Aldrich, Saint Louis, MO, USA). A final concentration of 2 µM was used for each primer, and 1 µL of DNA was used in each reaction. Real-time PCR was performed on a Mastercycler pro S instrument (Eppendorf, Hamburg, Germany) using the following program: 94 °C for 2 min, followed by 40 cycles of 94 °C for 15 s and 60 °C for 90 s. Relative quantity of DNA was estimated using Realplex software (Eppendorf, Hamburg, Germany).

### 4.4. Zeta Potential and Particle Size Analyses

Large scale binding mixture of DNA and magnetic micro- and nanoparticles was prepared in 1.5-mL Eppendorf tubes: 40 µL of DNA (~10^10^ copies/µL) mixed with 40 µL sodium acetate-HCl (0.75 M, pH = 6), 32 µL magnetic particles, and 240 µL of 85% ethanol. As control, zeta potential was measured prior and after the addition of DNA.

The zeta potential and particle size were measured in triplicates on Zetasizer MALVERN (Malvern Instruments Ltd., Malvern, UK), using the same absorption coefficient (10 for dispersive environment, 3 for dispersive phase), viscosity (0.8872 cP), equilibrating time (2 min), temperature (25 °C), and refraction index (1.333 for dispersive environment, 3.00 for dispersive phase). For particle size, an angle of 173° backscatter was used. Zeta potential analysis was based on the Smoluchowsky model to contemplate the decrease in particle concentration [F(κa) = 1.50]. The duration of analysis varied according to the number of runs, which was between 20 and 40. The instrument’s defaults were chosen for voltage settings and attenuation time. One cuvette was used for each measurement (type DTS1070 for zeta potential and type ZEN0040 for particle size).

## 5. Conclusions

Chemically coated magnetic particles are cheap and easy-to-synthesize multipurpose tools for the isolation of biomaterials. In this study, four magnetic particles were synthesized and screened for DNA binding. The particles were individually surface-coated with silica, PVP, phosphate and polyphosphate. All measurements showed that all particles were positive polycharged monodisperse systems.

Silica-coated MAN37 particles were 0.48 µm in size and exhibited highest mean zeta potential (+5.55 mV). Upon exposure to DNA in the binding solution, zeta potential decreased near to zero, but no visible change in the particle size was observed. After immobilization on magnet, including the washing of the particles with ethanol, and the elution of bound DNA, only 0.6% of theoretical amount of DNA was detected using quantitative PCR.

Polyvinylpyrrolidone-coated MAN127 particles were 2.97 µm in size and exhibited the second highest mean zeta potential (+2.46 mV). Upon exposure to DNA in the binding solution, zeta potential decreased near to zero, but no visible change in particle size was observed. After immobilization on magnet, the washing of the particles with ethanol, and the elution of bound DNA, approx. 166.9% of the total amount of DNA was detected using quantitative PCR. This overestimation indicates that the PCR process might have been enhanced due to the release of chemicals from the PVP coating material.

Phosphate-coated MAN158 particles were 3.67 µm in size with a mean zeta potential (+1.95 mV). Upon exposure to DNA in the binding solution, no visible change was observed in zeta potential or particle size. After immobilization on magnet, the washing of the particles with ethanol, and the elution of bound DNA, only 0.1% of the total amount of DNA was detected using quantitative PCR.

Tripolyphosphate-coated MAN164 particles were 2.93 µm in size with mean zeta potential (+1.78 mV). Upon exposure to DNA in the binding solution, zeta potential decreased below zero (−1.25 mV), while mean particle size increased to 4.81 µm. After immobilization on magnet, the washing of the particles with ethanol, and the elution of bound DNA, approx. 76.5% of the total amount of DNA was detected using quantitative PCR.

This study highlights the use of zeta potential and particle size analyses for the study of particle-DNA interaction. Here, these methods provided valuable information that can be supportive to quantitative PCR in the study of particle-DNA binding efficiency.

## Figures and Tables

**Table 1:** Summary of results: quantitative PCR, zeta potential, and particle size.

Analysis	MAN37 Silica-Coated	MAN127 PVP-Coated	MAN158 Phosphate-Coated	MAN164 Tripolyphosphate-Coated
qPCR analysis (copies ± SD) *	1.48 × 10^7^ ± 0.32 × 10^7^	4.17 × 10^9^ ± 1.68 × 10^9^	3.14 × 10^6^ ± 2.43 × 10^6^	1.91 × 10^9^ ± 0.32 × 10^9^
Binding percentage **	0.6%	166.9%	0.1%	76.5%
Zeta potential ζ (mV)	+5.55 ± 2.10	+2.46 ± 0.30	+1.95 ± 0.69	+1.78 ± 1.73
Zeta potential ζ in presence of DNA (mV)	−0.04 ± 0.31	−0.29 ± 0.34	+1.72 ± 0.73	−1.25 ± 1.75
Zeta potential ζ change	decreased	decreased	-	decreased
Particle size (µm)	0.48 ± 0.07	2.97 ± 0.18	3.67 ± 0.40	2.93 ± 0.55
Particle size in presence of DNA (µm)	0.44 ± 0.09	3.05 ± 0.77	3.82 ± 0.25	4.81 ± 0.73
Particle size change	-	-	-	increased

* Final DNA elution volume was 40 µL while 1 µL was used in each qPCR analysis. A factor of ×40 was used in calculation of total DNA amount retrieved; ** Binding percentage = total DNA amount retrieved/initial DNA amount × 100%. The initial amount of DNA was 10^11^ copies.

## Abbreviations

MAN37	silica-coated magnetic particles
MAN127	polyvinylpyrrolidone-coated magnetic particles
MAN158	phosphate-coated magnetic particles
MAN164	tripolyphosphate-coated magnetic particles
SEM	scanning electron microscopy
PVP	polyvinylpyrrolidone
